# [Corrigendum] Protective effects of metformin against myocardial ischemia-reperfusion injury via AMPK-dependent suppression of NOX4

**DOI:** 10.3892/mmr.2025.13752

**Published:** 2025-11-17

**Authors:** Yan Shi, Shu-Ai Hou

Mol Med Rep 24: 712, 2021; DOI: 10.3892/mmr.2021.12351

Following the publication of this paper, it was drawn to the Editor's attention by a concerned reader that a pair of the p-AMPK western blots shown in Fig. 3B were strikingly similar to AMPK blots shown in Fig. 5A; in addition, the AMPK blots shown in Fig. 4A were similar to the GAPDH blots shown in Fig. 5A, albeit the dimensions and intensities of the bands differed slightly comparing the two figure parts.

Upon re-examining their original data, the authors realized that inadvertent errors had been made in terms of the assembly of Figs. 3 and 4. The revised versions of Figs. 3 and 4, now showing the correct p-AMPK western blots in Fig. 3B and the AMPK blots in Fig. 4A, are shown on the next page. The authors wish to emphasize that these errors did not affect the results or the main conclusions reported in the study. All the authors approve of the publication of this corrigendum, and the authors are grateful to the Editor of *Molecular Medicine Reports* for allowing them the opportunity to publish this. The authors regret their oversight in allowing these errors to be included in the paper, and apologize to the readership for any inconvenience caused.

## Figures and Tables

**Figure 3.**
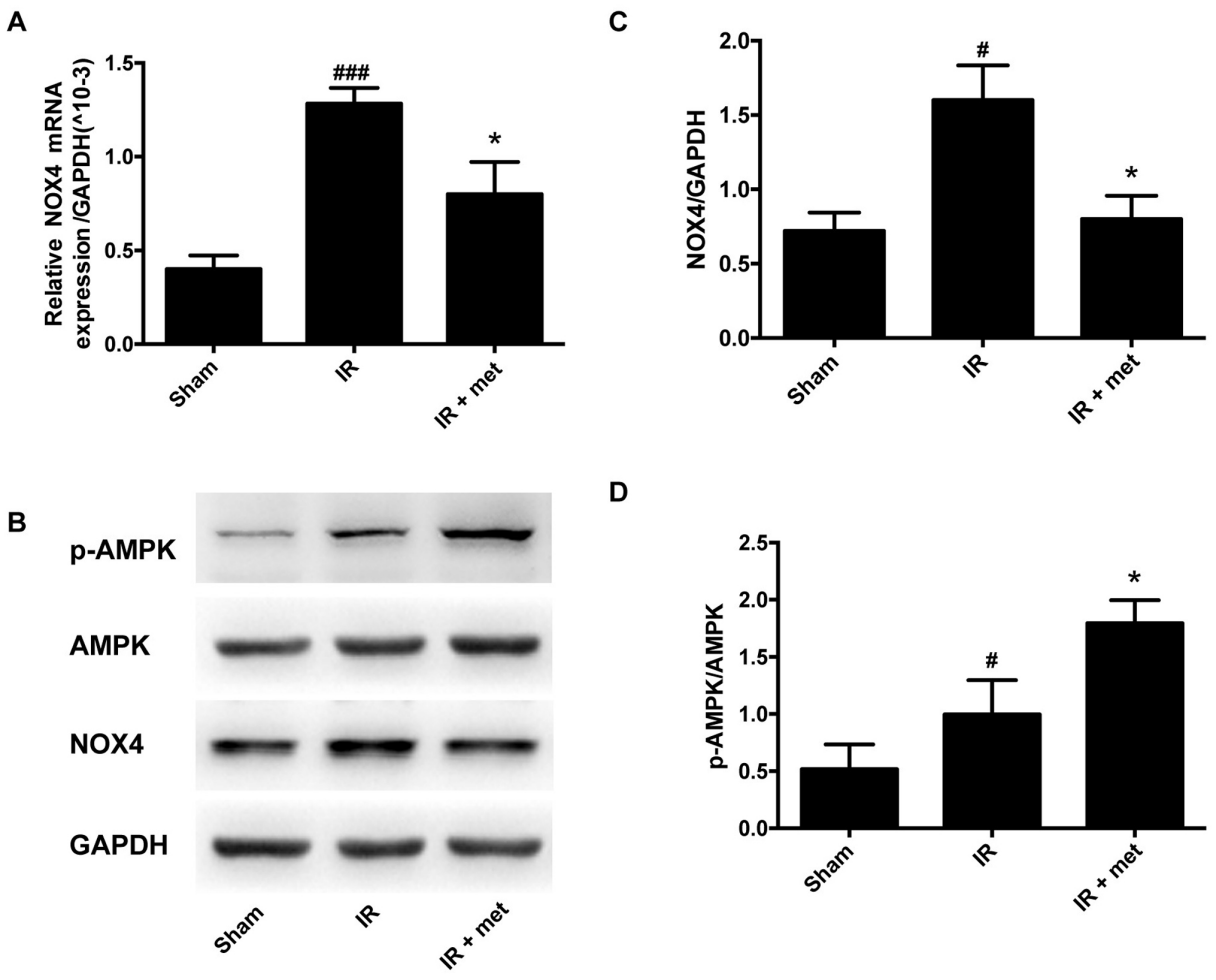
Effect of metformin on the activity of NOX4 and the activation of AMPK. (A) Expression of NOX4 mRNA as determined via reverse transcription-quantitative PCR. (B) Representative protein levels of p-AMPK, AMPK and NOX4 as determined via western blotting. (C) Semi-quantification of NOX4 protein levels. (D) Semi-quantification of the p-AMPK/AMPK ratio. Protein levels were normalized to GAPDH. Data are shown as the mean ± SEM (n=6). ^#^P<0.05, ^###^P<0.001 vs. Sham; *P<0.05 vs. IR. Sham, sham-operated control; IR, ischemia-reperfusion; met, metformin; NOX, NADPH oxidase; AMPK, adenosine 5’-monophosphate-activated protein kinase; p, phosphorylated.

**Figure 4.**
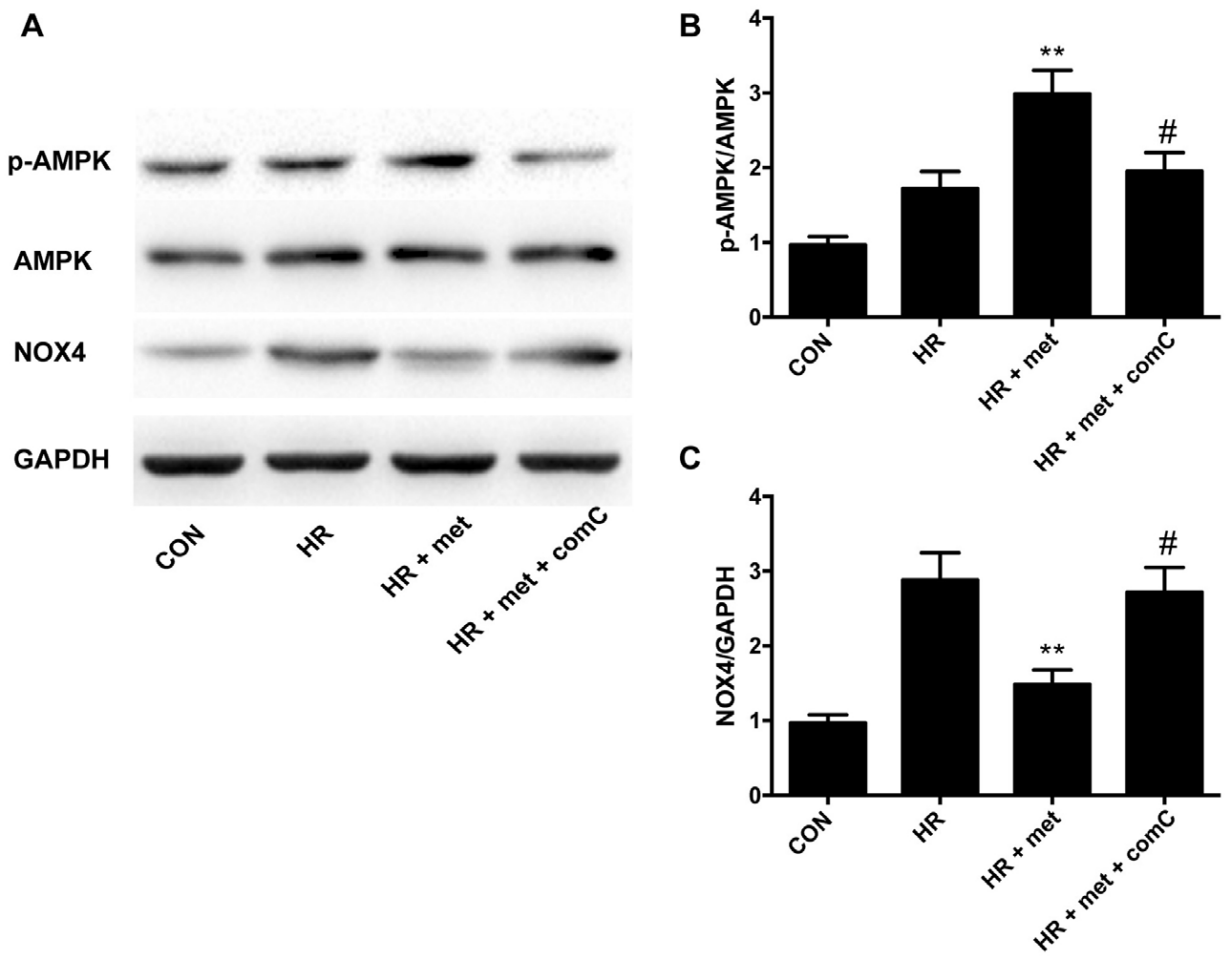
AMPK inhibitor upregulates the expression of NOX4 protein *in vitro.* (A) Representative protein levels of p-AMPK, AMPK and NOX4 as determined via western blotting. (B) Semi-quantification of the p-AMPK/AMPK ratio. (C) Semi-quantification of NOX4 protein levels. Protein levels were normalized to GAPDH. Data are shown as the mean ± SEM (n=6). **P<0.01 vs. HR; ^#^P<0.05 vs. HR + met. CON, control; HR, hypoxia-reoxygenation; met, metformin; comC, compound-C; NOX, NADPH oxidase; AMPK, adenosine 5’-monophosphate-activated protein kinase; p, phosphorylated.

